# Special Education of Children with Fetal Alcohol Spectrum Disorder

**DOI:** 10.1080/09362835.2015.1064415

**Published:** 2016-03-23

**Authors:** Svetlana Popova, Shannon Lange, Larry Burd, Seungree Nam, Jürgen Rehm

**Affiliations:** ^a^ Centre for Addiction and Mental Health; ^b^ University of Toronto; ^c^ North Dakota School of Medicine; ^d^ Technische Universität Dresden

## Abstract

The current study aimed to estimate the cost associated with special education among children (5 to 14 years) with Fetal Alcohol Spectrum Disorder (FASD) in elementary and middle school by sex, age group, and province and territory in Canada. It was estimated that there were 6,520 students with FASD receiving special education in Canada in 2011–2012. The cost of special education among these students was 53.5 million Canadian dollars. Implications for decision- and policymakers, educational systems and school staff are discussed.

Maternal alcohol consumption during pregnancy increases the risk for a wide range of adverse health outcomes, and is an established cause of Fetal Alcohol Spectrum Disorder (FASD). FASD is a nondiagnostic umbrella term used to characterize the full range of damage caused by prenatal alcohol exposure, varying from mild to severe and encompassing a broad array of deficits (Chudley et al., 2005). FASD includes three alcohol-related categorical diagnoses: Fetal Alcohol Syndrome (FAS), Partial Fetal Alcohol Syndrome (pFAS), and Alcohol-Related Neurodevelopmental Disorder (ARND; Chudley et al., 2005). FAS is the most severe and visibly identifiable form of FASD. Alcohol is a teratogen and therefore, prenatal alcohol exposure can adversely affect any organ or system of the fetus. As a result, children with FASD may have a broad array of congential anomalies, as well as cognitive, behavioral, emotional, and adaptive functioning deficits. These impairments are likely to have lifelong implications.

As children affected by prenatal alcohol exposure get older (as they approach adolescence), they are at an increased risk of developing secondary disabilities, such as school failure and dropout, mental health problems, sexually deviant behavior, alcohol and other drug problems, unemployment, homelessness, and involvement with the criminal justice system (Streissguth et al., 2004). The impairments caused by prenatal alcohol exposure result in significant social and economic costs to society (Abel & Sokol, 1987; Harwood, 2000; Legge, Roberts, & Butler, 2001; Lupton, Burd, & Harwood, 2004; Public Health Agency of Canada [PHAC], 2003, 2005; Popova et al., 2011, 2012, 2013a, 2013b, 2013c, 2014, 2015; Stade et al., 2009).

FASD is associated with learning and behavioral problems, developmental disabilities, and speech-language deficits, all of which increase the need for special education services and the risk of poor academic achievement if such services are not received. Kvigne and colleagues (2004) reported that among a sample of 78 children with FAS/pFAS, 74% of children with FAS had a developmental delay (40% of children with pFAS), 70% had a speech-language deficit (43%, pFAS), 23% had a learning disability (20%, pFAS), and 14% suffered from developmental disabilities (23%, pFAS). Additionally, Burd, Klug, Martsolf, and Kerbeshian (2003) reported that 35% of their sample of children with FASD had a learning disability, and 26% had a developmental delay.

Given the delays and deficits commonly experienced by children with FASD, the majority of these children require special education services. If such support is not provided, children with FASD are extremely likely to experience problems related to education and literacy. Children with FASD in the education system are most often recognized for their behavioral problems, and thus, are identified as difficult students to manage. Streissguth and colleagues (2004) reported that among a sample of individuals with FASD, 14% of 161 school children and 61% of 250 adolescents and adults had disrupted school experiences. Approximately 53% of the adolescents with FASD had been suspended from school, 29% had been expelled, and 25% had dropped out. The most frequently mentioned learning problems were attention deficits (70%) and repeatedly incomplete schoolwork (58%; Streissguth et al., 2004). Further, Kvigne and colleagues (2004) reported that among their sample of 78 children with FAS/pFAS, 16% of the children with FAS and 11.4% of children with pFAS reported school failures.

In a recent Canadian study, it was reported that children with FASD were more than three times as likely to repeat a grade, and more than nine times as likely to receive special education funding for special needs compared to the general population (Brownell et al., 2013). However, there are no data available on the proportion of children with FAS/FASD requiring and receiving special education and the associated cost in Canada.

The current modeling study was designed to (1) estimate the number of children with FAS/FASD receiving special education by sex, age group, and province and territory; and (2) estimate the cost associated with special education among children with FAS/FASD in Canada in 2011–2012. This is the first study to estimate the number of children with FAS/FASD receiving special education and the associated cost not only in Canada, but also in any other country of the world. All cost figures are presented in Canadian dollars.

The current study is part of a large economic project, with multiple components, working toward estimating the overall burden and cost associated with FASD in Canada (Popova et al., 2012b, 2013a, 2013b, 2013c, 2014, 2015).

## Method

A short survey was sent to the Minister of Education for each province and territory in January 2012 requesting the following information:

1. Number of children with FAS/FASD receiving special education in the province or territory;
2. Number of children with disabilities/special needs receiving special education in the province or territory; and
3. Cost of special education per child per year in the province or territory.

In this study, the estimate was restricted to elementary and middle school children (5 to 14 years) due to data availability issues pertaining to the number of children with disabilities receiving special education above the age of 14 years in Canada.

### Estimation of the total number of children in elementary and middle school in Canada

The total number of children in elementary and middle school by sex, age group (5 to 9 years and 10 to 14 years), and province and territory in Canada in 2011–2012 was obtained from Statistics Canada (2013).

### Estimation of the number of children with disabilities in elementary and middle school in Canada

In order to estimate the number of children with disabilities, the proportion of children with disabilities by sex and age group, obtained from the most recent *Participation and Activity Limitation Survey 2006: A Profile of Education for Children with Disabilities in Canada* (Statistics Canada, 2008), was applied to the total number of children in elementary and middle school in each Canadian province and territory in 2011–2012.

### Estimation of the number of children receiving special education in elementary and middle school in Canada

In order to estimate the number of children in Canada receiving special education, the proportion of children with disabilities receiving special education by sex and age group, obtained from the *Participation and Activity Limitation Survey 2006: A Profile of Education for Children with Disabilities in Canada* (Statistics Canada, 2008), was applied to the number of children with disabilities in each Canadian province and territory in 2011–2012.

### Estimation of the number of children with FAS/FASD receiving special education in elementary and middle school in Canada

Two strategies were employed in order to obtain the number of children with FAS/FASD receiving some form of special education in Canada. First, a comprehensive literature search on the prevalence of children with FAS/FASD receiving special education in Canada or in any other country was conducted. Second, as stated, a short survey was sent to the Minister of Education for each province and territory.

### Estimation of the cost of special education among children with FAS/FASD in elementary and middle school in Canada

There were two strategies employed in order to obtain the average cost of special education per child by province and territory. First, as stated, a short survey was sent to the Minister of Education for each province and territory. Second, a search of the readily available published reports found on the Ministry of Education website for each province and territory was conducted. Three provinces (Ontario, Manitoba, and Nova Scotia) provided the cost of special education per child via the survey, and the cost data for British Columbia and Prince Edward Island were obtained from the respective Ministry of Education websites.

In cases where the cost of special education per child was reported by funding levels, which are dependent on the severity of the disability, the lowest cost per child was used to obtain the most conservative estimate. It should be noted that there is considerable variation in the eligibility criteria used to determine the level of funding per-child across the country, and any funding comparisons must consider the factors that influence per-child funding levels in order to be meaningful. For example, level 2 funding in one province/territory may not always be lower than level 3 funding in another province/territory. Furthermore, when cost data were not available for a province or territory, the average overall cost of special education per child (estimated by averaging the cost of special education in the provinces and territories for which data were available) was used.

Thus, in order to estimate the cost of special education among children with FAS/FASD, the average estimated cost of special education per child for each province and territory was applied to the estimated number of children with FAS/FASD receiving special education by sex and age group for each respective province and territory. Table 1 presents the average cost of special education per child for each province and territory.

**Table 1.** The average cost of special education per child in elementary and middle school by province and territory in Canada in 2011–2012.

Province and Territory	Average Cost of Special Education per Child per Year
Alberta	$7,976^a^
British Columbia	$9,200^b^
Manitoba	$9,220^c^
New Brunswick	$7,976^a^
Newfoundland and Labrador	$7,976^a^
Northwest Territories	$7,976^a^
Nova Scotia	$5,564^d^
Nunavut	$7,976^a^
Ontario	$8,208^e^
Prince Edward Island	$7,761^f^
Quebec	$7,976^a^
Saskatchewan	$7,976^a^
Yukon	$7,976^a^

^a^ Based on the average overall cost of special education per child using data from British Columbia, Manitoba, Nova Scotia, Ontario, Prince Edward Island.

^b^ Based on level 3 funding (Category H: Intensive behaviour intervention or serious mental illness; British Columbia, Ministry of Education, 2011).

^c^ Based on level 2 funding (based on the student’s profile of need and level of support required for a major portion of the school day; personal communication, J. Blais, Director, Education Program and Student Services Branch, August 2012).

^d^ Based on 22,597 students receiving funding and a total grant of $125,734,200 (personal communication, R. Jennex, Minister of Education, August 2012).

^e^ Based on 307,020 students receiving funding (2009–2010) and a total grant of $2.52 billion (2012–2013; personal communication, B. Finlay, Director, Special Education Policy and Program Branch, July 2012).

^f^ Based on 309 students receiving funding (estimated in the current study) and a total of $2.4 million for special education funding (Prince Edward Island, Department of Education and Early Childhood Development, 2012).

## Results

The overall response rate from the Minister of education for each province and territory was 61.5% (8 out of 13: Alberta, British Columbia, Manitoba, Newfoundland and Labrador, Northwest Territories, Nova Scotia, Ontario, and Quebec). However, some of the provinces responded that the data were not collected/available, and only one province, Newfoundland and Labrador, provided FASD-specific data.

### Total number of children in elementary and middle school in Canada

According to Statistics Canada (2013), there were 3.7 million children aged 5 to 14 years (1.9 million boys and 1.8 million girls) in Canada in 2011–2012. There were 1.8 million children 5 to 9 years (928 thousand boys and 882 thousand girls) and 1.9 million children 10 to 14 years (987 thousand boys and 931 thousand girls) in Canada in 2011–2012.

The number of children in elementary and middle school by sex, age group, and province and territory is presented in Table 2.

**Table 2.** Number of children with FAS/FASD in elementary and middle school receiving special education and the associated sost by sex, age group, and province and territory in Canada in 2011–2012.

Province and Territory	5 to 9 Years			10 to 14 Years			Total		
	Boys	Girls	Both	Boys	Girls	Both	Boys	Girls	Both
**Alberta**									
Total number of children^a^	113,474	108,009	221,483	114,842	108,104	222,946	228,316	216,113	444,429
Number of children with one or more disabilities^b^	6,014	3,240	9,254	6,891	4,000	10,890	12,905	7,240	20,145
Number of children with disabilities receiving special education^c^	2,177	1,173	3,350	3,314	1,924	5,238	5,491	3,097	8,842
*Number of children with FAS receiving special education^d^*	*22*	*12*	*34*	*33*	*19*	*52*	*55*	*31*	*88*
*Cost of special education of children with FAS*	*$173,646*	*$93,557*	*$267,203*	*$264,352*	*$153,452*	*$417,804*	*$437,998*	*$247,009*	*$685,007*
**Number of children with FASD receiving special education^e^**	**196**	**106**	**302**	**298**	**173**	**471**	**494**	**279**	**773**
**Cost of special education of children with FASD**	**$1,562,818**	**$842,010**	**$2,404,829**	**$2,379,166**	**$1,381,072**	**$3,760,238**	**$3,941,984**	**$2,223,082**	**$6,165,066**
**British Columbia**									
Total number of children^a^	113,351	106,927	220,278	124,099	116,098	240,197	237,450	223,025	460,475
Number of children with one or more disabilities^b^	6,008	3,208	9,215	7,446	4,296	11,742	13,454	7,503	20,957
Number of children with disabilities receiving special education^c^	2,175	1,161	3,336	3,581	2,066	5,648	5,756	3,227	8,984
*Number of children with FAS receiving special education^d^*	*22*	*12*	*33*	*36*	*21*	*56*	*58*	*32*	*90*
*Cost of special education of children with FAS*	*$200,077*	*$106,833*	*$306,910*	*$329,498*	*$190,090*	*$519,588*	*$529,575*	*$296,923*	*$826,498*
**Number of children with FASD receiving special education^e^**	**196**	**105**	**300**	**322**	**186**	**508**	**518**	**290**	**809**
**Cost of special education of children with FASD**	**$1,800,695**	**$961,496**	**$2,762,191**	**$2,965,480**	**$1,710,810**	**$4,676,290**	**$4,766,175**	**$2,672,307**	**$7,438,481**
**Manitoba**									
Total number of children^a^	38,591	36,510	75,101	41,147	38,558	79,705	79,738	75,068	154,806
Number of children with one or more disabilities^b^	2,045	1,095	3,141	2,469	1,427	3,895	4,514	2,522	7,036
Number of children with disabilities receiving special education^c^	740	396	1,137	1,188	686	1,874	1,928	1,083	3,011
*Number of children with FAS receiving special education^d^*	*7*	*4*	*11*	*12*	*7*	*19*	*19*	*11*	*30*
*Cost of special education of children with FAS*	*$68,266*	*$36,557*	*$104,823*	*$109,488*	*$63,269*	*$172,757*	*$177,753*	*$99,826*	*$277,580*
**Number of children with FASD receiving special education^e^**	**67**	**36**	**102**	**107**	**62**	**169**	**174**	**97**	**271**
**Cost of special education of children with FASD**	**$614,390**	**$329,015**	**$943,404**	**$985,390**	**$569,423**	**$1,554,812**	**$1,599,779**	**$898,437**	**$2,498,216**
**New Brunswick**									
Total number of children^a^	18,648	17,612	36,260	20,794	19,096	39,890	39,442	36,708	76,150
Number of children with one or more disabilities^b^	988	528	1,517	1,248	707	1,954	2,236	1,235	3,471
Number of children with disabilities receiving special education^c^	358	191	549	600	340	940	958	531	1,498
*Number of children with FAS receiving special education^d^*	*4*	*2*	*5*	*6*	*3*	*9*	*10*	*5*	*15*
*Cost of special education of children with FAS*	*$28,537*	*$15,255*	*$43,792*	*$47,865*	*$27,107*	*$74,972*	*$76,402*	*$42,362*	*$118,764*
**Number of children with FASD receiving special education^e^**	**32**	**17**	**49**	**54**	**31**	**85**	**86**	**48**	**134**
**Cost of special education of children with FASD**	**$256,829**	**$137,299**	**$394,128**	**$430,786**	**$243,959**	**$674,745**	**$687,616**	**$381,258**	**$1,068,873**
**Newfoundland and Labrador**									
Total number of children^a^	12,894	12,280	25,174	13,871	13,245	27,116	26,765	25,525	52,290
Number of children with one or more disabilities^b^	683	368	1,052	832	490	1,322	1,516	858	2,374
Number of children with disabilities receiving special education^c^	247	133	381	400	236	636	648	369	1,017
*Number of children with FAS receiving special education^d^*	*2*	*1*	*4*	*4*	*2*	*6*	*6*	*4*	*10*
*Cost of special education of children with FAS*	*$19,731*	*$10,637*	*$30,368*	*$31,929*	*$18,801*	*$50,730*	*$51,661*	*$29,438*	*$81,099*
**Number of children with FASD receiving special education^e^**	**22**	**12**	**34**	**36**	**21**	**57**	**58**	**33**	**92**
**Cost of special education of children with FASD**	**$177,582**	**$95,732**	**$273,314**	**$287,364**	**$169,210**	**$456,574**	**$464,946**	**$264,942**	**$729,888**
**Northwest Territories**									
Total number of children^a^	1,450	1,435	2,885	1,456	1,428	2,884	2,906	2,863	5,769
Number of children with one or more disabilities^b^	77	43	120	87	53	140	164	96	260
Number of children with disabilities receiving special education^c^	28	16	43	42	25	67	70	41	111
*Number of children with FAS receiving special education^d^*	*0*	*0*	*0*	*0*	*0*	*1*	*1*	*0*	*1*
*Cost of special education of children with FAS*	*$2,219*	*$1,243*	*$3,462*	*$3,352*	*$2,027*	*$5,379*	*$5,570*	*$3,270*	*$8,840*
**Number of children with FASD receiving special education^e^**	**3**	**1**	**4**	**4**	**2**	**6**	**6**	**4**	**10**
**Cost of special education of children with FASD**	**$19,970**	**$11,187**	**$31,157**	**$30,164**	**$18,243**	**$48,407**	**$50,134**	**$29,430**	**$79,564**
**Nova Scotia**									
Total number of children^a^	22,702	21,507	46,057	25,598	23,950	49,548	48,300	45,457	93,757
Number of children with one or more disabilities^b^	1,203	645	1,848	1,536	886	2,422	2,739	1,531	4,270
Number of children with disabilities receiving special education^c^	436	234	669	739	426	1,165	1,174	660	1,843
*Number of children with FAS receiving special education^d^*	*4*	*2*	*7*	*7*	*4*	*12*	*12*	*7*	*18*
*Cost of special education of children with FAS*	*$24,235*	*$12,996*	*$37,230*	*$41,105*	*$23,716*	*$64,820*	*$65,339*	*$36,712*	*$102,051*
**Number of children with FASD receiving special education^e^**	**39**	**21**	**60**	**66**	**38**	**105**	**106**	**59**	**165**
**Cost of special education of children with FASD**	**$218,111**	**$116,961**	**$335,072**	**$369,941**	**$213,443**	**$583,384**	**$588,052**	**$330,404**	**$918,455**
**Nunavut**									
Total number of children^a^	1,760	1,650	3,410	1,645	1,533	3,178	3,405	3,183	6,588
Number of children with one or more disabilities^b^	93	50	143	99	57	155	192	106	298
Number of children with disabilities receiving special education^c^	34	18	52	47	27	75	81	45	126
*Number of children with FAS receiving special education^d^*	*0*	*0*	*1*	*0*	*0*	*1*	*1*	*0*	*1*
*Cost of special education of children with FAS*	*$2,693*	*$1,429*	*$4,123*	*$3,787*	*$2,176*	*$5,963*	*$6,480*	*$3,605*	*$10,085*
**Number of children with FASD receiving special education^e^**	**3**	**2**	**5**	**4**	**2**	**7**	**7**	**4**	**11**
**Cost of special education of children with FASD**	**$24,240**	**$12,863**	**$37,103**	**$34,079**	**$19,585**	**$53,664**	**$58,319**	**$32,448**	**$90,766**
**Ontario**									
Total number of children^a^	368,686	349,730	718,416	395,799	373,569	769,368	764,485	723,299	1,487,784
Number of children with one or more disabilities^b^	19,540	10,492	30,032	23,748	13,822	37,570	43,288	24,314	67,602
Number of children with disabilities receiving special education^c^	7,074	3,798	10,872	11,423	6,648	18,071	18,496	10,446	28,943
*Number of children with FAS receiving special education^d^*	*71*	*38*	*109*	*114*	*66*	*181*	*185*	*104*	*289*
*Cost of special education of children with FAS*	*$580,597*	*$311,743*	*$892,340*	*$937,573*	*$545,697*	*$1,483,269*	*$1,518,170*	*$857,440*	*$2,375,610*
**Number of children with FASD receiving special education^e^**	**637**	**342**	**978**	**1,028**	**598**	**1,626**	**1,665**	**940**	**2,605**
**Cost of special education of children with FASD**	**$5,225,375**	**$2,805,686**	**$8,031,061**	**$8,438,153**	**$4,911,272**	**$13,349,425**	**$13,663,528**	**$7,716,958**	**$21,380,487**
**Prince Edward Island**									
Total number of children^a^	3,738	3,698	7,436	4,330	4,067	8,397	8,068	7,765	15,833
Number of children with one or more disabilities^b^	198	111	309	260	150	410	458	261	719
Number of children with disabilities receiving special education^c^	72	40	112	125	72	197	197	113	309
*Number of children with FAS receiving special education^d^*	*1*	*0*	*1*	*1*	*1*	*2*	*2*	*1*	*3*
*Cost of special education of children with FAS*	*$5,566*	*$3,117*	*$8,683*	*$9,698*	*$5,617*	*$15,315*	*$15,264*	*$8,734*	*$23,998*
**Number of children with FASD receiving special education^e^**	**6**	**4**	**10**	**11**	**7**	**18**	**18**	**10**	**28**
**Cost of special education of children with FASD**	**$50,092**	**$28,051**	**$78,143**	**$87,283**	**$50,555**	**$137,838**	**$137,375**	**$78,606**	**$215,981**
**Quebec**									
Total number of children^a^	199,113	190,838	389,951	208,231	198,399	406,630	407,344	389,237	796,581
Number of children with one or more disabilities^b^	10,553	5,725	16,278	12,494	7,341	19,835	23,047	13,066	36,113
Number of children with disabilities receiving special education^c^	3,820	2,073	5,893	6,010	3,531	9,540	9,830	5,603	15,433
*Number of children with FAS receiving special education^d^*	*38*	*21*	*59*	*60*	*35*	*95*	*98*	*56*	*154*
*Cost of special education of children with FAS*	*$304,698*	*$165,303*	*$470,000*	*$479,321*	*$281,625*	*$760,947*	*$784,019*	*$446,928*	*$1,230,947*
**Number of children with FASD receiving special education^e^**	**344**	**187**	**530**	**541**	**318**	**859**	**885**	**504**	**1,389**
**Cost of special education of children with FASD**	**$2,742,279**	**$1,487,724**	**$4,230,003**	**$4,313,893**	**$2,534,626**	**$6,848,519**	**$7,056,172**	**$4,022,350**	**$11,078,523**
**Saskatchewan**									
Total number of children^a^	32,860	31,054	63,914	33,970	32,337	66,307	66,830	63,391	130,221
Number of children with one or more disabilities^b^	1,742	932	2673	2,038	1,196	3,235	3,780	2,128	5,908
Number of children with disabilities receiving special education^c^	630	337	968	980	576	1,556	1,611	913	2,524
*Number of children with FAS receiving special education^d^*	*6*	*3*	*10*	*10*	*6*	*16*	*16*	*9*	*25*
*Cost of special education of children with FAS*	*$50,285*	*$26,899*	*$77,184*	*$78,195*	*$45,902*	*$124,097*	*$128,479*	*$72,801*	*$201,280*
**Number of children with FASD receiving special education^e^**	**57**	**30**	**87**	**88**	**52**	**140**	**145**	**82**	**227**
**Cost of special education of children with FASD**	**$452,564**	**$242,089**	**$694,653**	**$703,752**	**$413,118**	**$1,116,870**	**$1,156,315**	**$655,207**	**$1,811,522**
**Yukon**									
Total number of children^a^	980	936	1,916	1,027	970	1,997	2,007	1,906	3,913
Number of children with one or more disabilities^b^	52	28	80	62	36	98	114	64	178
Number of children with disabilities receiving special education^c^	19	10	29	30	17	47	48	27	76
*Number of children with FAS receiving special education^d^*	*0*	*0*	*0*	*0*	*0*	*0*	*0*	*0*	*1*
*Cost of special education of children with FAS*	*$1,500*	*$811*	*$2,310*	*$2,364*	*$1,377*	*$3,741*	*$3,864*	*$2,188*	*$6,051*
**Number of children with FASD receiving special education^e^**	**2**	**1**	**3**	**3**	**2**	**4**	**4**	**2**	**7**
**Cost of special education of children with FASD**	**$13,497**	**$7,297**	**$20,794**	**$21,276**	**$12,392**	**$33,668**	**$34,773**	**$19,689**	**$54,462**
**CANADA (ALL Provinces/Territories)**									
Total number of children^a^	928,249	882,186	1,810,435	986,810	931,354	1,918,164	1,915,059	1,813,540	3,728,599
Number of children with one or more disabilities^b^	49,197	26,466	75,663	59,209	34,460	93,669	108,406	60,926	169,331
Number of children with disabilities receiving special education^c^	17,809	9,581	27,390	28,479	16,575	45,055	46,289	26,156	72,445
*Number of children with FAS receiving special education^d^*	*178*	*96*	*274*	*285*	*166*	*451*	*463*	*262*	*724*
*Cost of special education of children with FAS*	*$1,462,049*	*$786,379*	*$2,248,428*	*$2,338,525*	*$1,360,857*	*$3,699,382*	*$3,800,574*	*$2,147,235*	*$5,947,809*
**Number of children with FASD receiving special education^e^**	**1,603**	**862**	**2,465**	**2,563**	**1,492**	**4,055**	**4,166**	**2,354**	**6,520**
**Cost of special education of children with FASD**	**$13,158,442**	**$7,077,408**	**$20,235,851**	**$21,046,726**	**$12,247,709**	**$33,294,435**	**$34,205,168**	**$19,325,117**	**$53,530,285**

FAS = Fetal Alcohol Syndrome; FASD = Fetal Alcohol Spectrum Disorder.

^a^ Source: Statistics Canada (2013).

^b^ Based on disability rates of 5.3% for boys 5 to 9 years, 3.0% for girls 5 to 9 years, 6.0% for boys 10 to 14 years, and 3.7% for girls 10 to 14 years (Statistics Canada, 2008).

^c^ Based on rate of 36.2% for students with disabilities between the ages of 5 to 9 years, and 48.1% for students with disabilities between the ages of 10 to 14 years receiving some form of special education (Statistics Canada, 2008).

^d^ Based on a prevalence of 1% for FAS.

^e^ Based on a prevalence of 9% for FASD. *Note*. FASD figures are inclusive of those for FAS.

### Number of children with disabilities in elementary and middle school in Canada

The Participation and Activity Limitation Survey 2006 (Statistics Canada, 2008) revealed that 4.2% of Canadian children aged 5 to 9 years had one or more disabilities (5.3% among boys and 3.0% among girls) and 4.9% of Canadian children aged 10 to 14 years had one or more disabilities (6.0% among boys and 3.7% among girls).

Using these proportions, it was estimated that there were 169.3 thousand children with disabilities aged 5 to 14 years (108.4 thousand boys and 60.9 thousand girls) in Canada in 2011–2012. There were 75.7 thousand children with disabilities aged 5 to 9 years (49.2 thousand boys and 26.5 thousand girls) and 93.7 thousand children with disabilities aged 10 to 14 years (59.2 thousand boys and 34.5 thousand girls) in Canada in 2011–2012. The number of children with disabilities by sex, age group, and province and territory is presented in Table 2.

### Number of children with disabilities receiving special education in elementary and middle school in Canada

It was reported that in 2006, 43.1% of children with disabilities aged 5 to 14 years were receiving special education (36.2% of children 5 to 9 years and 48.1% of children 10 to 14 years; Statistics Canada, 2008).

Based on these proportions, it was estimated that there were 73.7 thousand children aged 5 to 14 years receiving special education (46.3 thousand boys and 26.2 thousand girls) in Canada in 2011–2012. There were 27.4 thousand children 5 to 9 years (17.8 thousand boys and 9.6 thousand girls) and 45.1 thousand children 10 to 14 years receiving special education (28.5 thousand boys and 16.6 thousand girls) in Canada in 2011–2012. The number of children receiving special education by sex, age group, and province and territory is presented in Table 2.

### Number of children with FAS/FASD receiving special education in elementary and middle school in Canada

The comprehensive literature search for publications reporting on the prevalence of children with FAS/FASD receiving special education in Canada did not produce any results.

The surveys, which were distributed to the Minister of Education for each province and territory, only yielded data from Newfoundland and Labrador regarding the number of children with FAS receiving special education. They reported that out of 11,342 students receiving special education in Newfoundland and Labrador in 2011–2012, 117 had FAS (Newfoundland and Labrador, Department of Education, n.d.). Therefore, the prevalence of FAS among children receiving special education was estimated to be approximately 1% (i.e., 10 per 1,000).

In Canada, the most commonly cited rough prevalence estimates are 1 per 1,000 for FAS and 9 per 1,000 for FASD (PHAC, 2003; Roberts & Nanson, 2000). Therefore, in order to estimate the number of students with FASD receiving some form of special education, a 1:9 (FAS:FASD) ratio was applied to the previously mentioned prevalence (i.e., 1% for FAS). This resulted in an estimate that 9% (i.e., 90 per 1,000) of students with FASD are receiving special education. These estimated proportions (1% for FAS and 9% for FASD) were assumed to be the same for each province and territory.

Based on a prevalence of 1%, it was estimated that there were 724 children with FAS 5 to 14 years of age receiving special education (463 boys and 262 girls) in Canada in 2011–2012. There were 274 children with FAS 5 to 9 years (178 boys and 96 girls) and 451 children with FAS 10 to 14 years receiving special education (285 boys and 166 girls) in Canada in 2011–2012.

Based on a prevalence of 9%, it was estimated that there were 6,520 children with FASD aged 5 to 14 years receiving special education (4,166 boys and 2,354 girls) in Canada in 2011–2012. There were 2,465 children with FASD 5 to 9 years (1,603 boys and 862 girls) and 4,055 children with FASD 10 to 14 years receiving special education (2,563 boys and 1,492 girls) in Canada in 2011–2012.

It is important to note that the figures reported for FASD include those for FAS. The number of children with FAS/FASD receiving some form of special education by sex, age group, and province and territory is presented in Table 2.

### Cost of special education among children with FAS/FASD in elementary and middle school in Canada

It was estimated that the cost of special education among children with FAS in elementary and middle school (i.e., children 5 to 14 years) was $5.9 million ($3.8 million among boys with FAS and $2.1 million among girls with FAS) in Canada in 2011–2012. The cost among children 5 to 9 years with FAS was $2.2 million ($1.5 million for boys with FAS and $786 thousand for girls with FAS), and the cost among children 10 to 14 years with FAS was $3.7 million ($2.3 million for boys with FAS and $1.4 million for girls with FAS).

Further, it was estimated that the cost of special education among children with FASD in elementary and middle school (i.e., children 5 to 14 years) was $53.5 million ($34.2 million among boys with FASD and $19.3 million among girls with FASD) in Canada in 2011–2012. The cost among children 5 to 9 years with FASD was $20.2 million ($13.2 million for boys with FASD and $7.1 million for girls with FASD), and the cost among children 10 to 14 years with FASD was $33.3 million ($21.0 million for boys with FASD and $12.2 million for girls with FASD).

It is important to note that the cost figures presented for FASD include those for FAS. The cost of special education among children with FAS/FASD in elementary and middle school by sex, age group, and province and territory are presented in Table 2.

## Discussion

Despite having used the most conservative approach, the estimated annual cost of special education among children with FASD in elementary and middle school in Canada is high. It was estimated that of the 169 thousand school children 5 to 14 years with a disability, 6,631 have FASD and are receiving some form of special education.

However, these estimates have important limitations that should be considered in the interpretation of this data. First, the number of children with FASD receiving special education in each Canadian province and territory was unavailable (with the exception of Newfoundland and Labrador, which, as stated, disclosed the number of children identified with FAS receiving special education). As such, the prevalence of children with FAS receiving some form of special education was estimated based on the data from Newfoundland and Labrador, which may not reflect the actual prevalence in the other provinces and territories.

Second, the prevalence of FASD among children receiving some form of special education was not available, and a 1:9 (FAS:FASD) ratio was assumed. This ratio is reflective of the most commonly cited prevalence among the general population of Canada (PHAC, 2003b; Roberts & Nanson, 2000) and aside from the fact that the true prevalence is currently unknown in Canada, it may not be reflective of the special education population.

Third, the cost of special education was only available for a few provinces and territories, and since special education funding is typically determined by level of need (i.e., severity of the deficits and disabilities), it is unknown whether the special education cost figures per student used in this study are reflective of students with FASD specifically.

Fourth, the proportion of children attending private schools, home schooled children, and those in residential care were not accounted for. The prevalence of FASD among these educational systems could be different than those that were estimated for the provincial and territorial educational systems.

Despite the fact that the estimated cost of special education among children with FASD is substantial, it is likely that it is still underestimated for the following reasons. First, it is likely that the estimated prevalence of children with FAS/FASD receiving some form of special education is underestimated given that FASD is largely underdiagnosed (Paintner et al., 2012). Second, the cost of special education for children with FASD was estimated for elementary and middle school only (due to data limitations), and thus, the cost of special education associated with students with FAS/FASD in high school was not included. Third, the cost of determining the eligibility for special education among children with FASD, which includes an assessment of their current skill level, their specific needs, and their functional abilities (Kalberg & Buckley, 2007), was also not included in these cost calculations. Finally, the costs of specialized training for teachers providing special education services and programs were not included.

Although this study has several limitations, it does provide a working estimate, which is a powerful tool for understanding the burden that FASD has on the educational system. Therefore, the cost data presented in this study should serve to provide a baseline for funding agencies and school systems. This study should be considered as a first step in an incremental process to improve data collection on the cost and utilization of special education services by children and families who are affected by FASD. When such data becomes available, then additional research can refine these estimates over time.

The current estimates provide both national and international decision- and policymakers alike with a clear perspective on the magnitude of the burden and cost of special education among children with FASD. This study is intended to draw attention to the impact of FASD on the educational system, and the need for (1) the collaboration of education and health care professionals in the assessment, diagnosis, and support of students with FASD, as classroom observations can provide valuable information when assessing a child; (2) teacher education programs and university faculties of education to develop courses on FASD; and (3) mandatory training in FASD for any teacher seeking a career in special education (Bredberg, 2011).

Further, the implications of this study for school staff include several areas of emphasis. First, clear guidelines or procedural protocols for teachers to use when designing special education programs for students with FASD need to be made readily available. Given that teachers and other support staff will undoubtedly meet a number of children with FASD within their teaching careers, there is an urgent need to develop and implement systematic training for teachers to better understand the needs of students with FASD and the best educational strategies when teaching them. However, as a spectrum disorder the deficits are complex and diverse, and additional research is needed in order to better understand the educational needs of students with FASD and the strategies that can be employed when teaching these students (Ryan & Ferguson, 2006). Second, given that FASD is largely underdiagnosed (Paintner et al., 2012), it is important that teachers are trained so that they can recognize potential cases of FASD. Teachers could then begin the process of initiating interventions and strategies that can improve the child’s learning and educational experience. Accordingly, teachers need to be informed of the range of interventions and strategies available to them so that they can tailor their educational programs accordingly. Edmonds and Crichton (2008) have shown that offering students with FASD individualized and customized education, along with counseling and life support, has proven to be effective. Third, teachers are in a unique position to educate young people (both girls and boys) about the detrimental effects of consuming alcohol while pregnant. The educational arena is a prime location for prevention initiatives to be implemented.

## Funding

This work was supported by the Public Health Agency of Canada.
